# Peas in Rouge: Tyrosine Supplementation Enhances RUBY Reporter Visibility in *Pisum sativum*

**DOI:** 10.3390/plants14243719

**Published:** 2025-12-05

**Authors:** Veronika Simonova, Elina Potsenkovskaia, Nikolai Kozlov, Alexandra Vanina, Elena Efremova, Kirill Smirnov, Anastasia Artemiuk, Anna Kiseleva, Anna Brynchikova, Zakhar Konstantinov, Varvara Tvorogova

**Affiliations:** 1 Plant Biology and Biotechnology Department, Sirius University of Science and Technology, 1 Olympic Avenue, 354340 Sochi, Russia; nikasimonova14@gmail.com (V.S.); anykisely@gmail.com (A.K.); krubaza@mail.ru (V.T.); 2 Komarov Botanical Institute of the Russian Academy of Sciences, Professora Popova Street, 2, 197376 Saint Petersburg, Russia; 3 Department of Genetics and Biotechnology, Faculty of Biology, Saint Petersburg State University, Universitetskaya nab., 7–9, 199034 Saint Petersburg, Russia; alexandraspb15@gmail.com (A.V.); elena.efremova@spbu.ru (E.E.); kirill.vad.smirnov@gmail.com (K.S.);; 4 All-Russia Research Institute for Agricultural Microbiology (ARRIAM), Pushkin, Podbelsky Chausse 3, 196608 St. Petersburg, Russia

**Keywords:** RUBY, *Pisum sativum*, pea, plant transformation, reporter system

## Abstract

Genome modification of legumes, peas in particular, is accompanied by significant challenges. Establishing a reliable reporter system to identify tissue that expresses foreign DNA may help to optimize and develop transformation protocols for these species. The RUBY system, based on the synthesis of red betalain from tyrosine, offers a convenient solution for monitoring the efficiency of transgene introduction. To evaluate the effectiveness of RUBY application in pea tissue culture, we combined agrobacterial transformation with an in vitro cultivation system, inducing callus development. Transformed explants demonstrated RUBY pigmentation, but it disappeared during cultivation. We hypothesized that this issue is caused by tyrosine depletion. To check this suggestion, we tested whether tyrosine supplementation could maintain RUBY coloring. In the later stages, pigmentation still could not be preserved. However, our modified conditions increased the percent of colored shoot apex explants during the early cultivation stages. Thus, it is likely that some explants transformed with the RUBY cassette do not synthesize a sufficient amount of betalain due to the deficit of endogenous tyrosine. In this case, adding exogenous tyrosine would enhance betalain production and improve the detectability of tissues containing the RUBY cassette. These data can be used for the optimization of RUBY application conditions for peas and other species.

## 1. Introduction

Genome modification of plants relies on several key aspects, which usually include methods for delivering the foreign DNA, a suitable genetic vector, and in vitro conditions for the selection of modified tissues and regeneration. Different species, as well as different varieties, lines, and cultivars belonging to the same species, often require specific transformation and regeneration conditions tailored to their specific needs. The method of selecting cells expressing foreign DNA can affect the efficiency of regeneration [1]; thus, the choice of selection strategy is a crucial step in the development of a transformation technique. Commonly, the selection of genetically modified cells/tissues involves the application of genes for resistance to selective agents (antibiotics, herbicides) and/or reporter genes, which enable the visual detection of transgene-containing tissue. In some cases, the use of reporter genes is preferable to resistance genes, as this does not require the addition of antibiotics and herbicides to the cultivation medium. Along with transformed tissue detection, reporter genes enable the visualization of gene expression, protein localization, and the assessment of transformation efficiency and stability.

Reporters include fluorescent proteins such as GFP, RFP, YFP, DsRED, and mCherry, demanding specific light sources for visualization [2]. Enzymatic reporters such as GUS [3] and luciferase [4] require the presence of X-Gluc and luciferin, respectively, for staining. Pigments, such as anthocyanins and betalains, are naturally synthesized in plants and are visible to the naked eye. In some cases, the enzymes involved in the synthesis of these compounds can be used as reporters. Betalains are pigments derived from the amino acid tyrosine that are produced by some species from the Caryophyllales group [5]. Transgenic betalain-producing plants can be engineered in other lineages through heterologous expression of corresponding biosynthesis genes [6,7]. Recently, the RUBY reporter system was developed, encoding a group of enzymes that convert the colorless amino acid tyrosine into bright-red betalain [8]. This system enables the visualization of transformed tissue in vivo without additional equipment or expensive chemical reagents. Betalain coloring enables the rapid identification and selection of transformants.

The RUBY system has been successfully applied in transformation protocols for both herbaceous and woody plants. For example, using this system, transgenic colored tobacco (*Nicotiana benthamiana*), arabidopsis (*Arabidopsis thaliana*) [9], wheat (*Triticum* spp.), and barley (*Hordeum vulgare*) plants [10] were obtained. The production of betalain-colored hairy roots in *Plukenetia volubilis* [9], *Vigna radiata* [11], and members of the *Crassulaceae* family [12] has also been described. RUBY has been used to evaluate the efficiency of transformation in *Cannabis sativa*, *A. thaliana* [13], *Raphanus sativus* [14], and *V. radiata* [11]. Moreover, this marker was employed for genome editing to detect tissues and plants containing the CRISPR/Cas9 cassette in *Glycine max* [15] and *R. sativus* [14].

In addition, RUBY was used to analyze the activity of various constitutive and tissue-specific promoters in transgenic tissues of *A. thaliana* and *Oryza sativa* [8]. Furthermore, the transformation of *Gossypium hirsutum* plants with a RUBY construct under a promoter specific for fiber-forming cells caused the formation of pink-colored cotton fibers [16]. Moreover, RUBY was used to assess the level of silencing in *N. benthamiana* leaves [17]. The possibility of applying RUBY for the identification of haploids in agricultural plants such as *Zea mays* and *Solanum lycopersicon* has also been demonstrated [18]. This marker was also used to detect the interaction between transcription factor and DNA [19], to detect protein–protein interactions [20], and to evaluate the efficiency of plant cotransformation with multiple vectors [21]. Furthermore, RUBY is capable of providing insight into the molecular interaction of parasitic plants with their host plants. When tomato and arabidopsis plants were infected with the transgenic parasitic plant *Cuscuta campestris* carrying the RUBY cassette, red pigment was detected outside the haustorium itself, in the host tissues [22].

RUBY mediates betalain pigmentation using the amino acid tyrosine as a substrate. Notably, in the model species *N. benthamiana* and *N. tabacum*, no negative effect of this reporter on plant viability, regeneration, or reproduction was detected [9,23]. Moreover, in wheat, a positive correlation was found between the presence of betalain coloration and resistance to the brown rust pathogen *Puccinia triticina* [10]. Similarly, transgenic *N. tabacum* plants synthesizing betalain demonstrated increased resistance to *Botrytis cinerea* [7]. However, in *Z. mays*, strongly colored plants with the RUBY cassette showed slowed growth, which was likely associated with a tyrosine deficiency [24].

The effectiveness of visual screening of transgenic tissues depends on the accumulation of a certain amount of betalain for coloration to appear [9]. For example, in *Crassulaceae*, the red pigment was poorly distinguishable on the callus in the first days after transformation, but accumulated over time, which was particularly noticeable in the mature root zone [12]. For *A. thaliana*, it took two days for sufficient pigment accumulation after transformation events. At the same time, *A. thaliana* plants obtained from transgenic seeds retained their red color throughout their life cycle [8]. In *Z. mays*, betalain accumulation was observed 2–3 days after transformation, varying greatly in different tissues and at different stages of development [24].

The successful application of a visual reporter like RUBY could be particularly valuable for genome modification of species where in vitro procedures are challenging. *Pisum sativum* (pea) is an important agricultural crop, whose genetic improvement is hampered by recalcitrance to in vitro transformation and regeneration [25]. Indeed, there are numerous protocols for pea transformation, but most of them are developed for a specific genotype and/or characterized by low efficiency. For example, papers on developing pea transformation protocols report an efficiency of up to 0.04 [26], 0.002, or 0.135 transgenic plants per explant [27]. More recently, a paper described a transformation system for peas with an efficiency of up to 0.47. Nevertheless, this parameter varied between genotypes and did not exceed 0.05 for some of them [28]. One recent study on pea genome editing, which included the generation of stable transformants, reported 0.5% efficiency for transformation, i.e., 1 transgenic shoot per 200 seed explants [29]. Therefore, the optimization of pea transformation remains challenging.

In this study, we evaluated the applicability of the RUBY system for *P. sativum* cell cultures obtained from shoot apices or seed axes. According to our results, agrobacterial transformation with a RUBY-containing vector led to bright-red betalain pigmentation of pea explants, although they demonstrated non-persistent pigmentation that disappeared during cultivation. Interestingly, adding exogenous tyrosine increased the percent of explants from shoot apices that had RUBY coloration during the early cultivation stages, although this coloration was also unstable. Nevertheless, our results suggest that in some cultivation systems, adding exogenous tyrosine to the cultivation medium can improve the detectability of tissues containing the RUBY cassette. These data can be used for the optimization of RUBY application conditions for peas and other species.

## 2. Results

### 2.1. The Applicability of RUBY in the Transformation of Pisum sativum

To check the possibility of RUBY usage in *Pisum sativum*, we developed a new system for introducing recombinant DNA into pea tissues. Our system was based on the AGROBEST transformation protocol [30] and two different cultivation protocols, which were initially designed for somatic embryogenesis induction in pea. The first protocol, including the cultivation of embryo axes on the MSB medium with 2.26 μM 2.4-D, was adapted from the method of Griga [31], with the exception that embryos from dry seeds were used as an explant source, instead of immature embryos. After 35 days of cultivation on the auxin-containing medium, developed calli were transferred to the hormone-free medium for the next 35 days. The second protocol was based on a study by Kysely and Jacobsen [32] and included cultivation of explants from shoot apices in the MSmod medium with 4 μM 2.4-D for 35 days. After cultivation on this medium, developed calli were transferred to the medium containing 4.4 μM BAP for 35 days.

**Figure 1:**
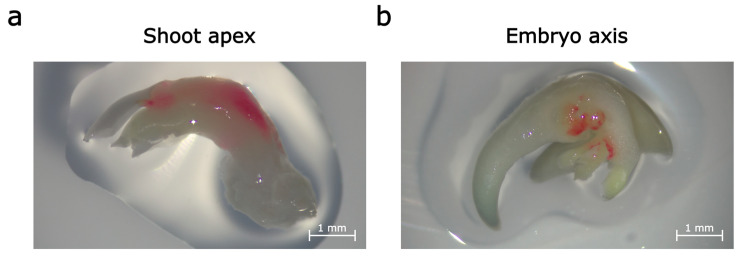
Explants from shoot apices (**a**) and embryo axes (**b**) transformed with 35S:RUBY plasmid on 7th day after agrobacterial infiltration.

We transformed explants from shoot apices and embryo axes with the construction 35S:RUBY [8] for RUBY cassette overexpression (Figure S1). After 3 days of co-cultivation and a further 4 days of cultivation without agrobacteria, transgenic explants showed red pigmentation (Figure 1). Calli developed from these explants had red pigment too, but not for a long time. Gradually, red coloration became paler, and some calli turned fully unpigmented.

### 2.2. Optimization of Cultivation Conditions for RUBY Visualization in Pisum sativum Explants

The RUBY reporter encodes a set of enzymes that convert tyrosine to betalain. We supposed that adding tyrosine to the cultivation medium could make the red coloration of transgenic calli more stable. As mentioned above, the protocols we used involved two major stages: 35 days of cultivation on media with auxin 2.4-Dichlorophenoxyacetic acid (2.4-D) and 35 days of cultivation on the medium with cytokinin 6-Benzylaminopurine (BAP-6) or hormone-free medium. To evaluate the possible effect of tyrosine on RUBY staining, we tested two different tyrosine concentrations (2.5 mM and 5 mM), either throughout the entire cultivation period or only during the second cultivation step (the last 35 days of cultivation).

**Figure 2:**
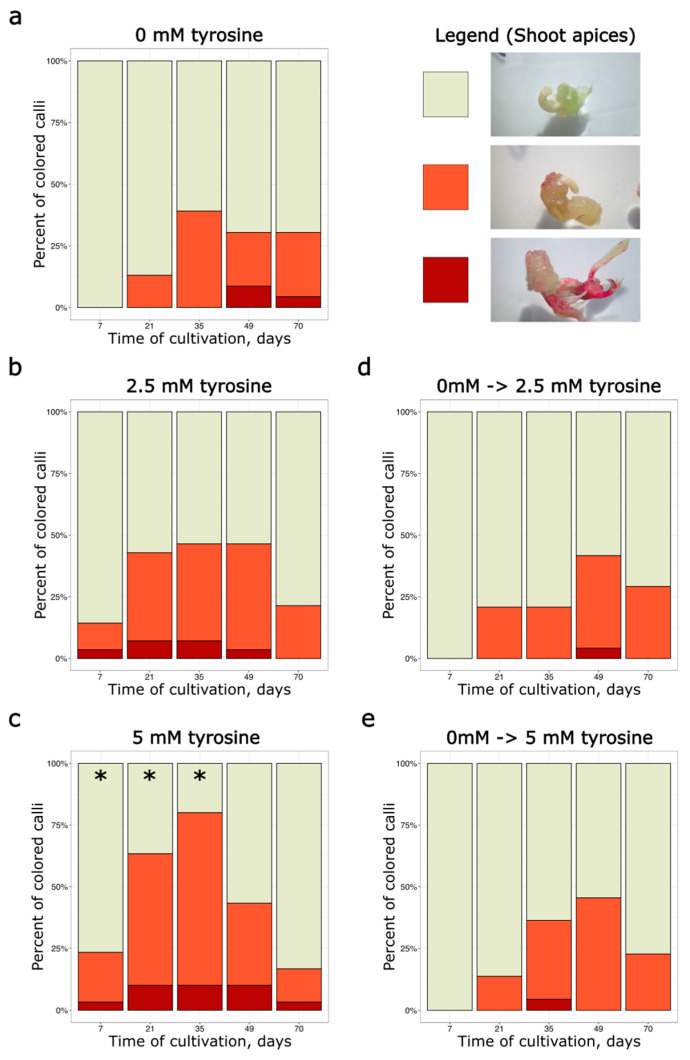
Percentage of transformed explants from shoot apices, which demonstrated different coloration coverages during the cultivation period. Explants were divided into 3 categories: beige—no staining; orange—spotty pigmentation; dark red—widespread pigmentation. (**a**) Control (tyrosine-free media). (**b**) Media with 2.5 mM tyrosine added throughout the entire 70-day cultivation period. (**c**) Media with 5 mM tyrosine added throughout the entire 70-day cultivation period. (**d**) Media with 2.5 mM tyrosine added only during the final 35 days of cultivation. (**e**) Media with 5 mM tyrosine added only during the final 35 days of cultivation. For each variant of tyrosine supplementation, 22–30 explants were used, which were observed throughout the whole cultivation period. Asterisks mark significant differences (*p*-value < 0.05, Fisher’s exact test) in the frequency of stained explants between the marked group and the control group of explants on tyrosine-free medium at the same stage of cultivation.

For transformed explants from shoot apices, the number of stained calli was significantly higher on 5 mM tyrosine-supplemented media compared to the control on the 7th, 21st and 35th day of cultivation (Figure 2, Table S1). For the medium with 2.5 mM tyrosine, no statistically significant effect was detected compared to the control. Nevertheless, the staining began to disappear on all calli, regardless of the culture medium. Furthermore, tyrosine supplementation that began after 35 days of cultivation had no detectable effect on staining (Figure 2a,d,e). No significant differences were observed between medium conditions on days 49 and 70 (Figure 2a–c).

**Figure 3:**
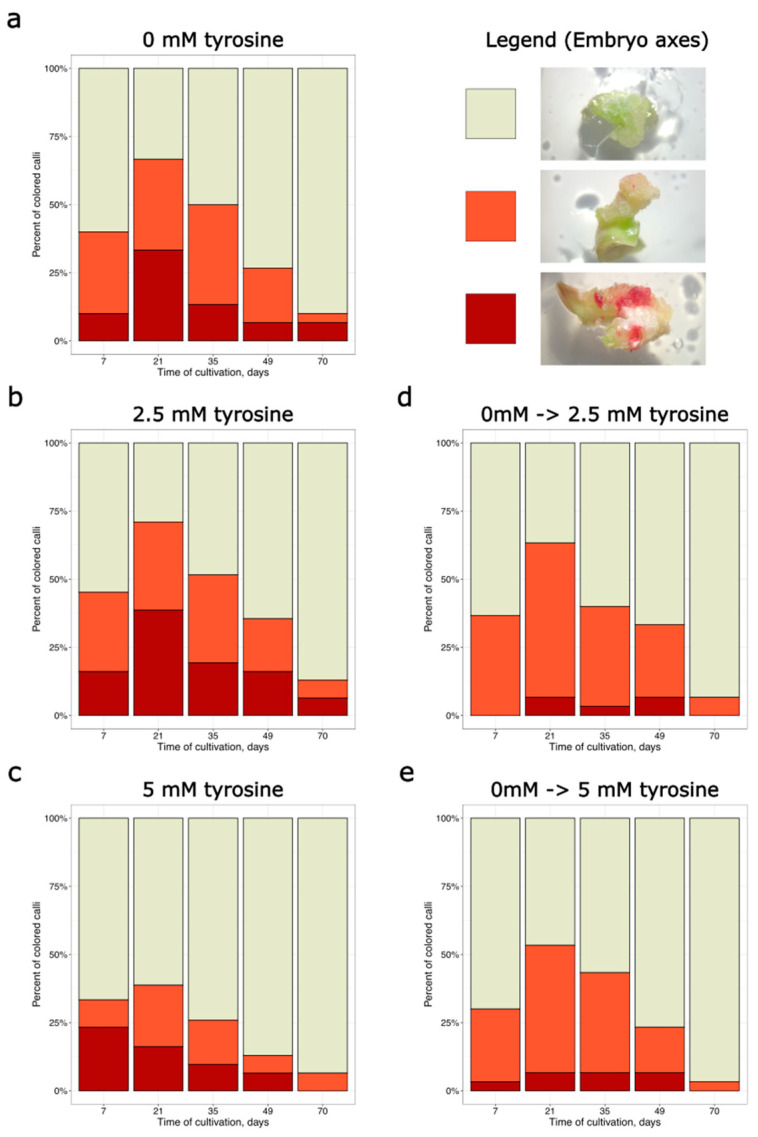
Percentage of transformed explants from embryo axes, which demonstrated different coloration coverages during the cultivation period. Explants were divided into 3 categories: beige—no staining; orange—spotty pigmentation; dark red—widespread pigmentation. (**a**) Control (tyrosine-free media). (**b**) Media with 2.5 mM tyrosine added throughout the entire 70-day cultivation period. (**c**) Media with 5 mM tyrosine added throughout the entire 70-day cultivation period. (**d**) Media with 2.5 mM tyrosine added only during the final 35 days of cultivation. (**e**) Media with 5 mM tyrosine added only during the final 35 days of cultivation. For each variant of tyrosine supplementation, 30–31 explants were used, which were observed throughout the whole cultivation period.

For transformed embryo axes, exogenous tyrosine in the medium did not have the same effect as for shoot apices. A slight decrease in the number of stained calli was observed at the highest tyrosine concentration used (5 mM), but no significant differences were found between five variants of cultivation, at all time points, according to Fisher’s exact test (Figure 3, Table S1).

### 2.3. Testing of Tyrosine Effect on Callus Phenotype

To check if exogenous tyrosine has any effect on calli development and viability, we cultured non-transformed shoot apices and embryo axis explants on the same media as the transformed explants for the same period (3 days on the 2.4-D-containing co-cultivation medium, then 32 days on the 2.4-D-containing callus induction medium, and then 35 days on the medium with cytokinin (BAP-6) or hormone-free medium for embryo axes and shoot apices, respectively) with the same tyrosine concentrations as we used previously (2.5 and 5 mM). After 70 days of cultivation, calli were weighted to estimate their growth rate, and their appearance was evaluated. We supposed that friable green or yellow callus tissue is more healthy and vigorous than dense yellow or brown tissue. Therefore, we divided calli into four phenotypic categories according to their color and structure: calli containing predominantly friable green tissue; calli containing friable green and yellow tissue; calli containing friable yellow tissue and some dense green tissue; and calli containing yellow or brown tissue without green tissue.

**Figure 4:**
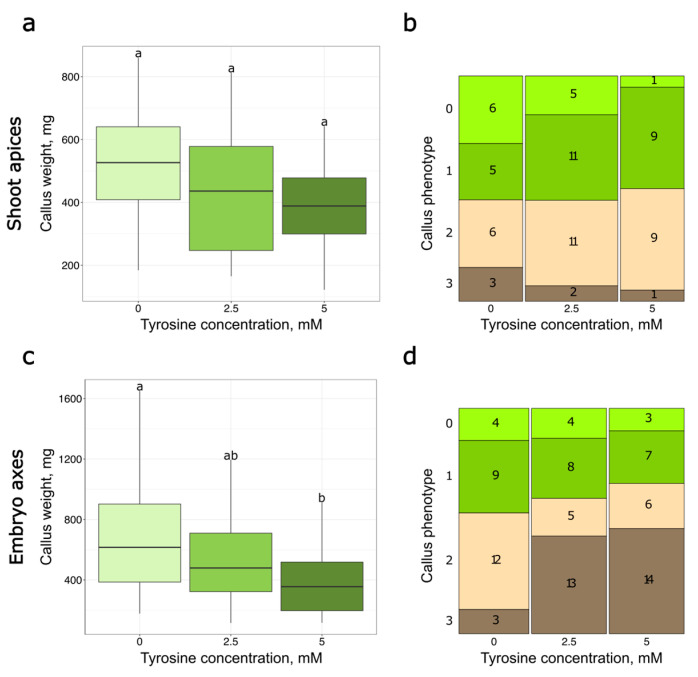
Weight of calli developed from shoot apices (**a**) and embryo axes (**c**) from explants cultured with different tyrosine concentrations. To assess the statistical significance of the observed differences, the Kruskal–Wallis test with the post hoc Dunn test and Holm’s *p*-value adjustment was used. Different lowercase letters represent values with statistically significant differences (*p*-value < 0.05). Light green–calli cultivated on tyrosine-free medium, green–calli cultivated on medium with 2.5 mM tyrosine, dark green–calli cultivated on medium with 5 mM tyrosine. Mosaic plot representing the number of calli developed from shoot apices (**b**) and embryo axes (**d**) explants belonging to specific phenotypic groups: 0—contains predominantly friable green tissue; 1—contains friable green and yellow tissue; 2—contains friable yellow tissue and some dense green tissue; 3—contains yellow or brown tissue without green tissue.

As a result, the groups of calli obtained from the shoot apices, cultured at three different tyrosine concentrations (with tyrosine 0 mM, 2.5 mM and 5 mM), did not have significant differences in either weight (according to Kruskal–Wallis test) or phenotype (according to Fisher’s exact text) (Figure 4a,b, Table S2). Similar analysis of explants from embryonic axes showed that explants cultivated on 5 mM tyrosine-containing medium developed calli with a lower weight (Figure 4c). Moreover, visual assessment revealed that after cultivation on the media with 2.5 mM or 5 mM tyrosine, a significantly higher fraction of explants developed brown or yellow calli (according to Fisher’s exact text), which suggests lower viability (Table S2).

### 2.4. Agrobacterial Co-Cultivation Duration Effect on Transgenic Callus Staining

**Figure 5:**
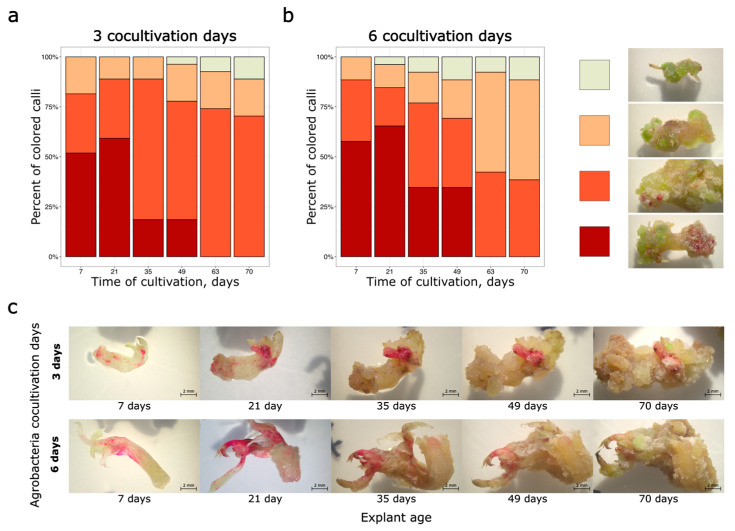
Percentage of transformed explants from shoot apices, which demonstrated different coloration coverages during the cultivation period after 3 (**a)** or 6 (**b**) days of co-cultivation with agrobacteria. Explants were divided into 4 categories: pale yellow-green—no staining; apricot—spotty pigmentation; orange-red—patchy pigmentation; dark red—widespread pigmentation. 27 or 33 explants were analysed after 3 or 6 days of co-cultivation, respectively. (**c**) Pictures of two individual calli after 3 or 6 days of co-cultivation on the 7th, 21st, 35th, 49th, and 70th day of cultivation.

According to the results presented above, 5 mM tyrosine supplementation significantly improved the detection of transformed shoot apex explants containing the RUBY expression cassette. We tested the possibility of optimized RUBY reporter system usage for transformation protocol optimization in peas. To investigate the influence of co-cultivation duration on the effectiveness of primary transformation (the percentage of foreign DNA containing calli with stained tissues), we transformed explants from shoot apices using the 35S:RUBY vector and co-cultivated the explants with *A. tumefaciens* for either 3 or 6 days. All media contained 5 mM tyrosine, since this medium composition allowed more explants expressing the reporter cassette to be detected. According to the analysis of RUBY staining, no differences in transformation effectiveness were detected between the different co-cultivation periods (Figure 5).

Interestingly, in this experiment, the transformation efficiency reached 100%. Initially, all calli exhibited some degree of pigmentation. However, similarly to our previous experiments, the pigmentation disappeared over time. Nevertheless, no statistically significant differences were found in the frequency of colored calli between the groups subjected to the three-day and six-day co-cultivation periods (Figure 5). Moreover, by the 70th day of cultivation, 7 out of 30 explants in the 6-day co-cultivation group had become infected with agrobacteria and been discarded. In contrast, no agrobacterial contamination was found in the three-day co-cultivation group. Based on our results, we recommend a three-day co-cultivation period to prevent *Agrobacterium* overgrowth without compromising transformation efficiency.

## 3. Discussion

Plant transformation systems require a marker to identify successfully transformed cells. For this purpose, a gene of resistance to antibiotics or herbicides and/or a reporter that can visualize tissue containing foreign DNA are delivered during transformation together with the target gene. In the present study, we tested the possibility of using RUBY, a vital marker of transformed tissue, as a reporter in *P. sativum* transformation. According to our results, transformation with the RUBY-containing vector allowed colored tissue to be obtained; however, transformed explants did not maintain pigmentation throughout the whole cultivation period.

Similar results were obtained for other plant species. For example, transformation of *C. campestris* with a vector containing the RUBY marker was described, after which 100% of explants showed red staining on the 5th day after inoculation with agrobacteria. However, over time, the number of stained explants significantly decreased [22]. Another study showed the disappearance of betalain staining after the transformation of *Phyllostachys* spp. seedlings, which was apparently due to the lack of integration of the transgene into the chromosome and its subsequent loss [33].

However, there are also examples of stable betalain coloration provided by the RUBY cassette. For example, it was reported that in *A. thaliana*, plants obtained from transgenic seeds after floral dip transformation retained red coloration throughout their life cycle, when *RUBY* was expressed under the control of the CaMV 35S constitutive promoter [8]. Stable transmission of RUBY coloration to subsequent generations was also observed in soybean [15].

We can suggest three possible reasons for RUBY coloration disappearing during tissue culture: lack of stable genome integration, transcriptional silencing of transgenes, or metabolic changes, for example, a lack of tyrosine, rendering betalain synthesis impossible.

Stable genome integration of a transgene is usually a desirable outcome of transformation. Nevertheless, the delivery of agrobacterial T-DNA into plant cells is supposed to be a much more frequent event than the following integration of a transgenic insert into the plant genome. Therefore, transient expression of transgenes in transformed cells can be observed [34]. Protocols describing short-term transgene expression have been described for different plant species, for example, sunflower [35] and arabidopsis [36]. Moreover, protocols for the expression of transgenes in transformed explant tissues usually report rather high efficiency, whereas the formation of transgenic regenerants from such explants is much less effective [37,38]. For peas, a transformation protocol was described, allowing production of callus tissue expressing recombinant DNA, from which plants were obtained; nevertheless, a transgenic insert was not identified in these regenerants [39]. Thus, the lack of stable integration of the reporter cassette could be the cause of RUBY coloration disappearance in the current study.

The cause of the instability in the explant coloration may be not only the loss of the transgene insert, but also a decrease in its expression level, which is a common issue for transgenic plants [40], as reviewed in [41,42].

In addition, a decrease in the amount of betalain may be associated with the metabolic features of the explant tissues. Betalain synthesis from the amino acid tyrosine occurs in several stages, during which four intermediate components are formed, such as l-3,4-dihydroxyphenylalanine (l-DOPA), cyclo-DOPA, betalamic acid, and betanidine [43]. The involvement of any of these substances in any other metabolic pathway in the cell can interfere with betalain synthesis and accumulation. At the same time, the metabolism features of explant cells can change during cultivation. Interestingly, in *Amaranthus tricolor*, which naturally synthesizes betalain, the content of this substance in cells can vary due to treatment with various growth regulators, such as 2,4-D, kinetin, BAP, and GA3. The likely reason for such an effect may be an alteration in the expression levels of biosynthesis genes, but metabolic changes also cannot be excluded [44].

We suggested that the addition of tyrosine, a betalain precursor, to the medium could increase the stability of staining if metabolic changes are the primary cause of coloration disappearing. However, the presence of tyrosine in the medium did not maintain the stability of betalain staining throughout the cultivation period. We can suggest that the depletion of exogenous tyrosine may also occur, leading to coloration instability, but this cause seems unlikely: the tyrosine-containing medium (MSB for embryo axis explants or MSmod for shoot apex explants) was renewed after 35 days of cultivation, during the transfer from callus induction medium to the auxin-free medium. The fresh medium contained 2.5 mM or 5 mM tyrosine. However, we did not observe a subsequent increase in the frequency of red-colored calli following this renewal (Figure 2b,c and Figure 3b,c). Moreover, we also used a separate cultivation scheme, in which tyrosine was added only to auxin-free medium after the initial 35 days of cultivation on the medium without tyrosine. In this case, no increase in coloration was observed (Figure 2d,e, and Figure 3d,e). Therefore, our results do not support the hypothesis that tyrosine depletion during cultivation could be a major cause of coloration disappearance.

Thus, we can suggest that loss of coloration is mediated by suppression of expression or loss of the transgene insert. Analysis of other reporter systems, along with molecular analysis, will allow us to check these suggestions. To verify the integration of T-DNA, Southern blot analysis is the gold-standard method [45]. However, the obtained colored calli are highly heterogenous, making Southern blot unfeasible. The next step is the development of a protocol for plant regeneration from calli obtained from this transformation system; such plants will be subjected to Southern blot analysis to check for stable integration. Specific PCR methods, such as TAIL-PCR [46], Wristwatch PCR [47], and other techniques, can be used to distinguish between stable T-DNA insertion and its transient expression, even in callus tissue. Simultaneously, the presence of T-DNA in plant cells can be verified by PCR with control reactions to exclude the possibility of the presence of residual agrobacterial DNA. The copy number of T-DNA in developing callus tissue can be evaluated with qPCR or ddPCR [48]. Finally, qPCR analysis of *RUBY* gene expression dynamics during calli cultivation can be used to determine if foreign DNA expression disappears together with RUBY coloration.

Nevertheless, even transient recombinant DNA expression can be used for pea genome modification. For example, in the case of CRISPR/Cas9-assisted genome editing, the integration of Cas9 and/or gRNA transgenes into the genome is not needed and may be undesirable. At the same time, maximization of the amount of tissue expressing the editing cassette is important, even if such expression is transient.

Still, exogenic tyrosine increased the number of stained explants after the transformation of shoot apices in the early stages of cultivation. At the same time, no similar effect of exogenous tyrosine was detected for another type of explant (embryo axes). We have no evidence to suggest that tyrosine supplementation enhances transformation efficiency. Instead, it is likely that in a tyrosine-free medium, some transgenic explants containing the reporter cassette do not synthesize a sufficient amount of betalain for detection due to the lack of tyrosine. The positive effect of tyrosine specifically on the staining of shoot apex explants suggests that tyrosine is present in their tissues in a limited amount, in contrast to the explants of embryo axes, which are seed fragments and probably contain a sufficient amount of tyrosine. These assumptions were also confirmed in experiments assessing the viability of non-transformed explants on a medium with different tyrosine contents: the addition of tyrosine to the medium had a negative effect on the development of embryonic axis explants, probably due to its excessive concentration. At the same time, this effect was absent in the case of shoot apex explants.

Thus, in the case of a limited amount of tyrosine in plant tissues, the addition of this amino acid to the culture medium may allow a larger number of explants containing transgenic tissue to be identified. Perhaps the system of culturing plant tissues with the RUBY cassette in the medium with tyrosine proposed in this study will also be useful for transforming other plant species. For example, for hairy roots of *P. volubilis*, *N. benthamiana*, and transgenic *A. thaliana* plants with RUBY, an insufficient degree of coloring was noted [9]. For maize (*Z. mays* L.) plants containing the RUBY cassette under the 35S promoter, coloring was also not observed in all tissues and organs [24].

In this work, we established the first successful application of the RUBY reporter system for *P. sativum*, an agriculturally important crop. Furthermore, we have shown, for the first time, that the application of exogenous tyrosine can enhance betalain-based RUBY coloration. These findings can be used for developing both transient expression and genome modification protocols for peas and other species.

## 4. Materials and Methods

### 4.1. Plant Material

In all experiments, the pea cultivar “Jaguar” was used, developed by the Federal State Budgetary Scientific Institution “Federal Scientific Center of Legumes and Groat Crops” (Oryol, Russia). Pea seeds were kindly provided by the Plastilin company (Moscow, Russia).

### 4.2. Bacterial Strains and Plasmids

For pea transformation, the *Rhizobium radiobacter* (*Agrobacterium tumefaciens*) AGL1 strain was used, containing the plasmid 35S:RUBY [8]. T-DNA contained the RUBY cassette under an enhanced 35S promoter and transcription terminator from the *A. thaliana* heat shock protein 18.2 gene [49], as well as the hygromycin B resistance gene under the control of the nopaline synthase gene promoter and terminator (Figure S1). The plasmid was introduced into *A. tumefaciens* through the freeze–thaw method [50].

### 4.3. Plant Transformation

#### 4.3.1. Explant Preparation

Seed sterilization was performed as follows: dry pea seeds were submerged in 96% sulfuric acid and incubated for 10 min with slow rotation. Then, sulfuric acid was removed, and seeds were rinsed 5–7 times with sterile water. After that, seeds were incubated in commercial bleach (Belizna), containing 5–7% sodium hypochlorite, for 10 min with slow rotation. Then, bleach was removed and seeds were rinsed again with sterile water 5–7 times. For explant preparation from embryo axes, sterilized seeds were left in a Petri dish partially submerged in sterile water for 1–2 days in the dark at room temperature. Then, in sterile conditions, imbibed seeds were cut into two halves along the plane dividing the cotyledons. The coat was removed, and from each half of a seed, the whole remaining part of the embryo axis was excised together with a small cotyledon part. For the preparation of explants from shoot apices, freshly prepared sterile seeds were left in a Petri dish partially submerged in sterile water for 5–7 days in the dark at room temperature. Then, in sterile conditions, shoot apices (about 3–5 mm in length) were cut from seedlings.

#### 4.3.2. Agrobacterium Preparation

The AGL1 strain with the 35S:RUBY plasmid [8] was cultivated in liquid and solid YEP medium (per 1 L:5 g NaCl, 10 g tryptone, 10 g yeast extract, 15 g agar (for solid medium)) with the addition of rifampicin (40 mg/L) and spectinomycin (100 mg/L). The strain was stored at −80 °C in the glycerol stock (500 μL of liquid overnight stationary-phase culture grown at 30 °C and 200 rpm in YEP with antibiotics mixed with 500 μL of sterile 50% glycerol). Before transformation, the strain was plated from glycerol stock on the solid YEP medium with rifampicin and spectinomycin and cultivated for 1–2 days at 30 °C. After that, bacteria from the solid medium were sown into 5 mL of liquid YEP with rifampicin and spectinomycin and cultivated overnight in the thermoshaker (30 °C, 200 rpm). The next day, 2 mL of night culture was added to the 250 mL flask with 50 mL of liquid AB-MES medium (AB salts, AB buffer, 10.66 g/L MES*1H_2_O, 20 g/L glucose, pH = 5.5) [30], alongside 200 μM of acetosyringone (AS), rifampicin (40 mg/L), and spectinomycin (100 mg/L), and cultivated in the thermoshaker (30 °C, 200 rpm) for 3 h. Then, the OD_600_ was measured, and the culture was centrifuged at 4000× *g* and room temperature for 15 min. After that, the supernatant was removed and bacteria were resuspended in 30 mL of the infiltration medium (liquid ABM-MSB or ABM-MSmod; see below) to the final OD_600_ of 0.1.

#### 4.3.3. Agrobacterial Transformation and Further Cultivation

The transformation method was based on the AGROBEST protocol [30]. For embryo axis transformation, liquid ABM-MSB medium (MS salts 0.5×, 13.9 mg/L FeSO_4_*7H_2_O, 18.9 mg/L Na_2_EDTA*2H_2_O, B5 vitamins 0.5×, AB salts 0.5×, AB buffer 0.5×, 10 g/L glucose, 15 g/L sucrose, 50 mg/L myo-inositol, 5.33 g/L MES*1H_2_O, pH = 5.5) with 200 μM AS and 2.3 μM 2.4-D was used for infiltration, containing 1/2 AB-MES [30] and 1/2 MSB medium [31]. For the transformation of shoot apices, liquid ABM-MSmod medium (MS salts 0.5×, 13.9 mg/L FeSO_4_*7H_2_O, 18.9 mg/L Na_2_EDTA*2H_2_O, 0.25 mg/L nicotinic acid, 0.25 mg/L pyridoxine HCl, 1 mg/L thiamine, MS vitamins 0.5×, AB salts 0.5×, AB buffer 0.5×, 10 g/L glucose, 15 g/L sucrose, 125 mg/L myo-inositol, 5.33 g/L MES*1H_2_O, pH = 5.5) with 200 μM AS and 4 μM 2.4-D was used for infiltration, containing 1/2 AB-MES [30] and 1/2 MSmod medium [32].

Explants (embryo axes or shoot apices) were added to the infiltration medium with resuspended agrobacteria and incubated at room temperature for 15 min with gentle agitation. After that, the medium was removed and explants were put onto co-cultivation media: solid ABM-MSB with 200 μM AS, 2.3 μM 2.4-D, and different tyrosine concentrations (for embryo axes) or solid ABM-MSmod with 200 μM AS, 4 μM 2.4-D, and different tyrosine concentrations (for shoot apices) with 0.7% agar. Explants were cultivated in the dark for 3 days unless otherwise stated. Then, explants were rinsed from bacteria 4 times in sterile water and, finally, rinsed in sterile water with cefotaxime (250 mg/L). After rinsing, explants were put onto media for callus induction in the light (16 light/8 dark). For embryo axes, MSB medium (MS salts, 27.8 mg/L FeSO_4_*7H_2_O, 37.8 mg/L Na_2_EDTA*2H_2_O, B5 vitamins, 30 g/L sucrose, 50 mg/L myo-inositol, 7 g/L agar, pH = 5.8) with 2.3 μM 2.4-D, 250 mg/L cefotaxime, and different tyrosine concentrations was used for callus induction. For shoot apices, MSmod medium (MS salts, 27.8 mg/L FeSO_4_*7H_2_O, 37.8 mg/L Na_2_EDTA*2H_2_O, 0.5 mg/L nicotinic acid, 0.5 mg/L pyridoxine HCl, 2 mg/L thiamine, 7 g/L agar) with 4 μM 2.4-D, 250 mg/L cefotaxime, and different tyrosine concentrations was used for callus induction. Despite the presence of the hygromycin B resistance gene in the 35S:RUBY vector, hygromycin B was not used for cultivation, because, according to our results, its addition does not increase the frequency of pea calli expressing recombinant DNA in this system [51]. Explants were cultivated in callus induction medium for 32 days. Then they were transferred to the hormone-free MSB medium with 250 mg/L cefotaxime and different tyrosine concentrations (for embryo axes) or to the MSmod medium with 4.4 μM BAP, 250 mg/L cefotaxime, and different tyrosine concentrations (for shoot apices). In this medium, the explants were cultivated for 35 days. In total, the cultivation period lasted for 70 days, starting from the day of transformation. Evaluation of betalain coloring caused by the RUBY cassette was performed on the 7th, 21st, 35th, 49th, and 70th day after transformation.

Common solutions used in media included MS salts (final concentrations: 1.9 g/L KNO_3_, 0.332 g/L CaCl_2_, 0.37 g/L MgSO_4_*7H_2_O, 1.65 g/L NH_4_NO_3_, 0.17 g/L KH_2_PO_4_) [52], B5 vitamins (final concentrations: 1 mg/L nicotinic acid, 1 mg/L pyridoxine HCl, 10 mg/L thiamine) [53], AB salts (final concentrations: 1 g/L NH_4_Cl, 0.3 g/L MgSO_4_*7H_2_O, 0.15 g/L KCl, 15 mg/L CaCl_2_, 2.5 mg/L FeSO_4_*7H_2_O), and AB buffer (final concentrations: 3 g/L K_2_HPO_4_, 0.992 g/L NaH_2_PO_4_) [30]. For the preparation of the solid cultivation media, L-tyrosine (Mister Prot, Stavropol, Russia) was used. L-tyrosine was added to the medium as the last component under constant stirring. The mixture was heated until the tyrosine was completely dissolved and then cooled to room temperature. The pH of the medium was measured and adjusted if necessary, after which agar was added. L-tyrosine powder was stored in a dark place at room temperature. All experiments with non-transformed explants included the same cultivation stages as for transformed explants, excluding the incubation of explants in the infiltration medium with agrobacteria and explants rinsing with water and cefotaxime.

### 4.4. Statistical Analysis and Software Used in the Study

Statistical analysis was performed in the R environment [54] using Rstudio version 2025.05.1+513 [55]. The R packages *dplyr *(version 1.1.4), *ggplot2* (version 4.0.0) [56], *multcompView *(version 0.1.10), *vcd* (version 1.4-13), and *stringr *(version 1.5.2) were used. For diagram drawing, Inkscape software version 1.4.2 was used.

## Data Availability

The original contributions presented in this study are included in the article/supplementary material. Further inquiries can be directed to the corresponding author.

## Supplementary Materials

The following supporting information can be downloaded at https://www.mdpi.com/article/10.3390/plants14243719/s1: Figure S1: Map of the 35S:RUBY vector used for transformation in the study. The following features are highlighted: bom—basis of mobility region from pBR322 [57]; CaMV 35S promoter (enhanced)—cauliflower mosaic virus 35S promoter with a duplicated enhancer region [58]; CYP76AD1—coding sequence for P450 oxygenase CYP76AD1, part of the RUBY cassette [8]; DODA—coding sequence for l-DOPA 4,5-dioxygenase, part of the RUBY cassette [8]; GT—coding sequence for glucosyltransferase, part of the RUBY cassette [8]; HygR—coding sequence for hygromycin B phosphotransferase, conferring resistance to hygromycin [59]; HSP-terminator—transcription terminator from the *A. thaliana* heat shock protein 18.2 gene [49]; LB T-DNA repeat—left border T-DNA repeat from nopaline C58 T-DNA; NOS promoter—nopaline synthase gene promoter [60]; NOS terminator—nopaline synthase gene terminator; ori—high-copy-number ColE1/pMB1/pBR322/pUC origin of replication [61]; P2A—sequence encoding 2A peptide from porcine teschovirus-1 polyprotein, part of the RUBY cassette [8]; pVS1 oriV—origin of replication for the Pseudomonas plasmid pVS1 [62]; pVS1 RepA—coding sequence for replication protein from the Pseudomonas plasmid pVS1 [62]; pVS1 StaA—stability protein from the Pseudomonas plasmid pVS1 [62]; RB T-DNA repeat—right border T-DNA repeat from nopaline C58 T-DNA; RUBY—RUBY cassette [8]; SmR—cds for aminoglycoside adenylyltransferase, conferring resistance to spectinomycin and streptomycin [63]; Table S1: Results of evaluation of differences in fractions of stained calli between media with different tyrosine supplementation. For statistical analysis, explants were divided into two groups: stained calli, demonstrating at least some pigmentation, and non-stained calli without any pigmentation. To assess the statistical significance of differences, Fisher’s exact test was used. *p*-value adjustment for multiple comparisons was performed according to the Bonferroni method. tyr0—control (tyrosine-free media); tyr2.5—media with 2.5 mM tyrosine added throughout the entire 70-day cultivation period; tyr5—media with 5 mM tyrosine added throughout the entire 70-day cultivation period; tyr0->2.5—media with 2.5 mM tyrosine added only during final 35 days of cultivation; tyr0->5—media with 5 mM tyrosine added only during final 35 days of cultivation; Table S2: Results of evaluation of differences in fractions of dense brown or yellow calli between media with different tyrosine supplementation. For statistical analysis, explants were divided into two groups: brown or yellow calli and all other types of calli. To assess the statistical significance of differences, Fisher’s exact test was used. *p*-value adjustment for multiple comparisons was performed according to the Bonferroni method. tyr0—control (tyrosine-free media); tyr2.5—media with 2.5 mM tyrosine added throughout the entire 70-day cultivation period; tyr5—media with 5 mM tyrosine added throughout the entire 70-day cultivation period.

## Abbreviations

The following abbreviations are used in this manuscript:

GFP	Green Fluorescent Protein
RFP	Red Fluorescent Protein
YFP	Yellow Fluorescent Protein
DsRED	Discosoma striata red fluorescent protein
GUS	β-glucuronidase
CRISPR	Clustered Regularly Interspaced Short Palindromic Repeats
BAP	6-Benzylaminopurine
2.4-D	2.4-Dichlorophenoxyacetic acid
GA3	Gibberellic Acid 3
DOPA	Dihydroxyphenylalanine
AS	Acetosyringone
TAIL-PCR	Thermal Asymmetric Interlaced Polymerase Chain Reaction
ddPCR	Droplet Digital Polymerase Chain Reaction
