# Supplementary figures and images for: Peas in Rouge: Tyrosine Supplementation Enhances RUBY Reporter Visibility in Pisum sativum
Source: Plants (Basel). 2025 Dec 5;14(24):3719. doi: 10.3390/plants14243719 (PMC12736525; doi:10.3390/plants14243719)

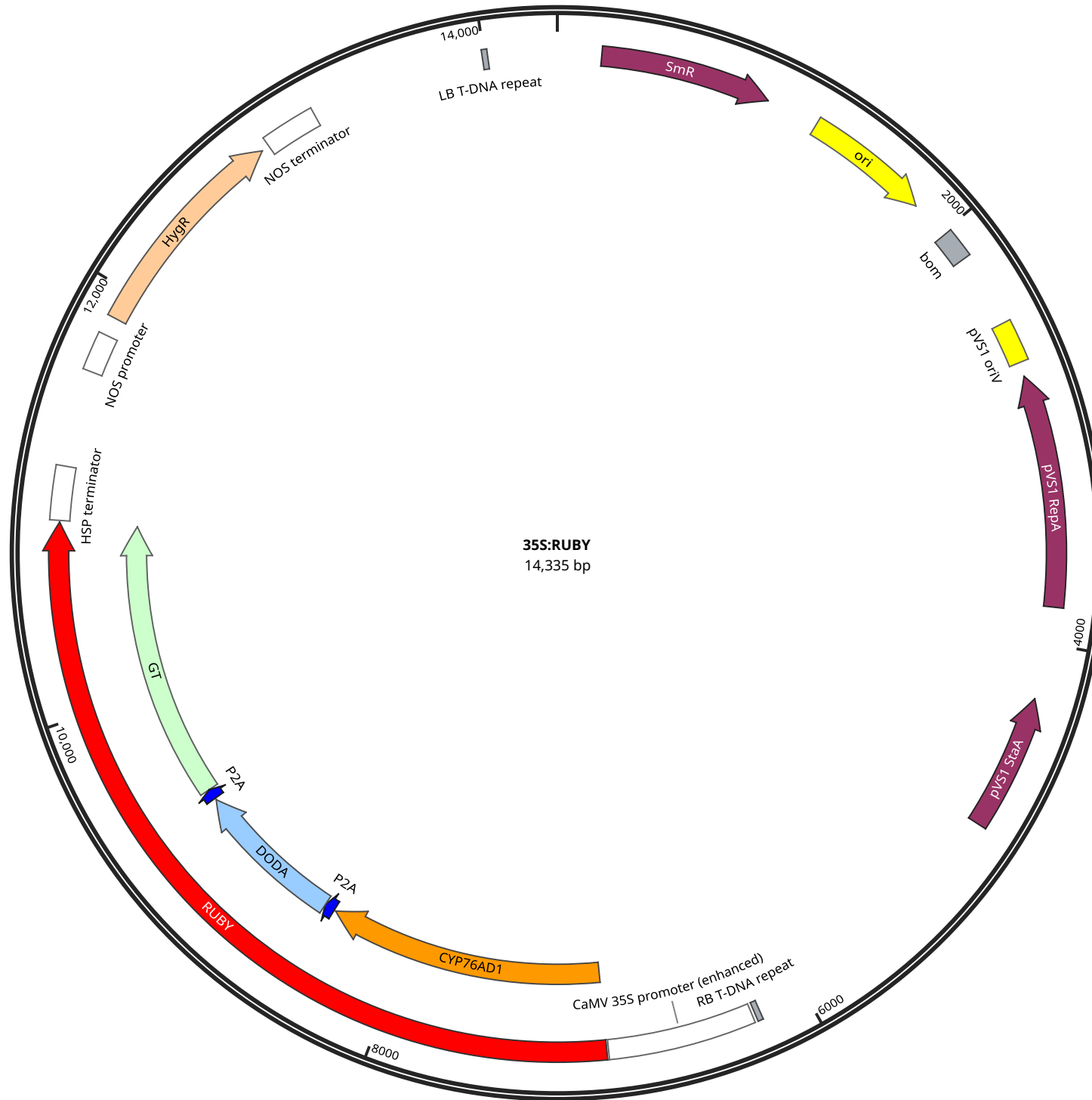

Supplement: Supplementary file 1 [file plants-14-03719-s001.zip › Figure S1.pdf]
